# Synchronous Bilateral Primary Testicular Tumors With Discordant Histopathology

**DOI:** 10.7759/cureus.20619

**Published:** 2021-12-22

**Authors:** Louisa Liu, Crystal Wang, Sameer Shah, Dina Saba, Neil Dudheker, Mary D Le, Vishal Ranpura

**Affiliations:** 1 Internal Medicine, University of California, Riverside School of Medicine, Riverside, USA; 2 Hematology and Oncology, Baylor University, Waco, USA; 3 Hematology and Oncology, University of California, Riverside School of Medicine, Riverside, USA; 4 Hematology and Oncology, University of California San Diego, San Diego, USA; 5 Pathology, Kaiser Permanente Riverside Medical Center, Riverside, USA; 6 Hematology and Oncology, Suburban Hematology Oncology, Lawrenceville, USA

**Keywords:** bilateral testicular masses, discordant histopathology, bilateral primary synchronous tumors, testicular cancer, testicular germ cell tumors

## Abstract

Concomitant presentation of histologically distinct bilateral testicular tumors is exceedingly rare. Here we report the case of a 20-year-old male who presented with a left testicular mass. He was found to have bilateral testicular masses on ultrasound and underwent bilateral orchiectomy. Left testicular pathology revealed a mixed germ cell tumor consisting of teratoma, seminoma, and germ cell neoplasia in situ; right testicular pathology revealed two foci of pure seminomas. He is currently on active surveillance and remains in remission at 18-month follow-up. Our case demonstrates the rare occurrence of bilateral primary synchronous testicular tumors with different histopathology in each testis. Despite the rarity of this condition, its treatment is based on standard management of unilateral testicular carcinoma, with the added element of prioritization of one tumor over the other. It is important for clinicians to tailor management for bilateral testicular germ cell tumors according to the most aggressive component.

## Introduction

Testicular carcinoma is the most common solid malignancy in men between the ages of 15 and 35 years. The incidence of testicular carcinoma in the United States is estimated to be six cases per 100,000 men per year [1]. Approximately 2-3% of testicular tumors are bilateral, the majority of which are metachronous [1]. Bilateral synchronous testicular tumors make up 10% of bilateral tumors and typically present with shared histological patterns in both testes, with bilateral seminoma as the most common presentation [2]. Discordant histological presentations of bilateral testicular tumors are thus exceedingly rare, with fewer than 100 cases described in the literature [2-8]. Here we contribute an additional case of bilateral primary synchronous testicular tumors with different histopathology in each testis.

## Case presentation

**Figure 1:**
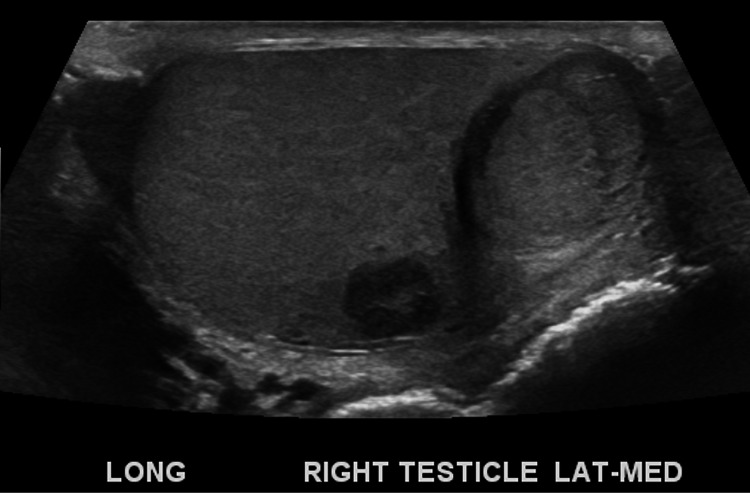
Right Testicular Masses on Ultrasound

**Figure 2:**
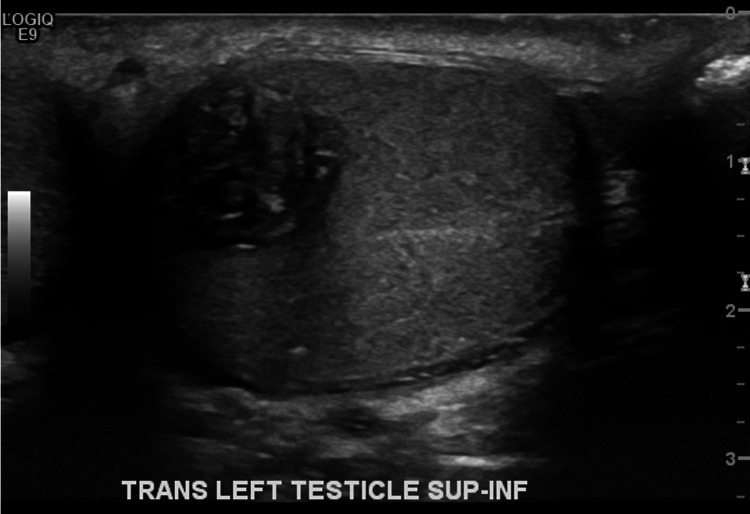
Left Testicular Mass on Ultrasound

A 20-year-old healthy male noticed a left scrotal mass on self-examination of testis in December 2019. Scrotal ultrasound revealed bilateral testicular masses. Two masses were identified in the right testis (Figure 1) measuring 2.19 cm X 1.82 cm X 1.66 cm mass in the mid-inferior portion and 1.02 cm X 0.82 cm x 0.95 cm mass in the mid-lateral portion. Both masses revealed internal and peripheral vascularity. In the left testis, there was a 1.86 cm X 1.09 cm X 1.42 cm testicular mass on the mid-portion of the left testis (Figure 2). The mass appeared complex with internal and peripheral vascularity, multiple cystic structures within, and calcifications. His tumor markers were measured, with alpha-fetoprotein (AFP) 5.5 nanogram (ng)/milliliter (mL), human chorionic gonadotrophin (HCG) <1 milli international unit (mIU)/mL and lactate dehydrogenase (LDH) 188 international units (IU)/liter (L).

**Figure 3:**
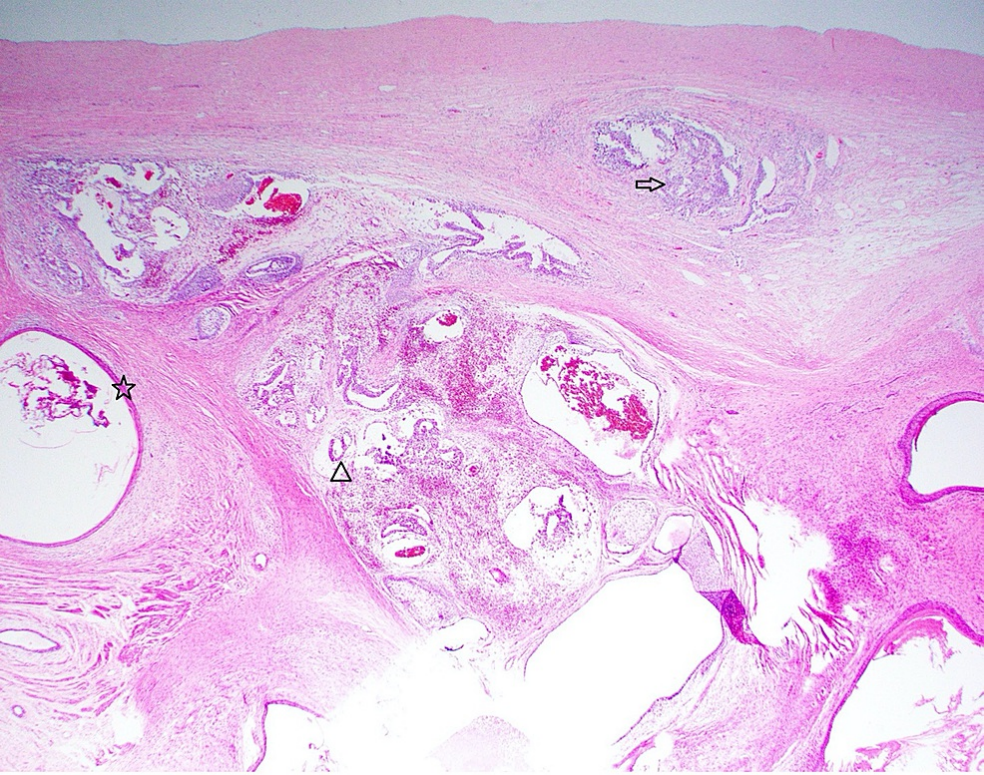
Left Testicular Tumor Histology H&E stain of the patient’s left testicular tumor shows a mixed germ cell tumor composed of post pubertal teratoma (star), embryonal carcinoma (arrow), and yolk sac tumor (triangle).

**Figure 4:**
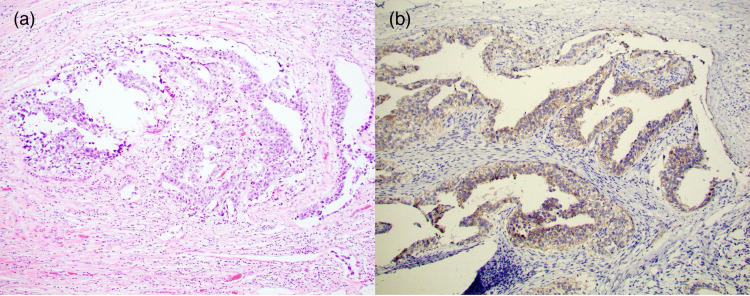
Embryonal Carcinomatous Component in Left Testicular Tumor (a) H&E stain of the embryonal carcinomatous component in the patient’s mixed testicular germ cell tumor in the left testis showing large anaplastic pleomorphic tumor cells. {b) Immunohistochemical stain showing CD30 expression on a section of the embryonal carcinomatous component.

**Figure 5:**
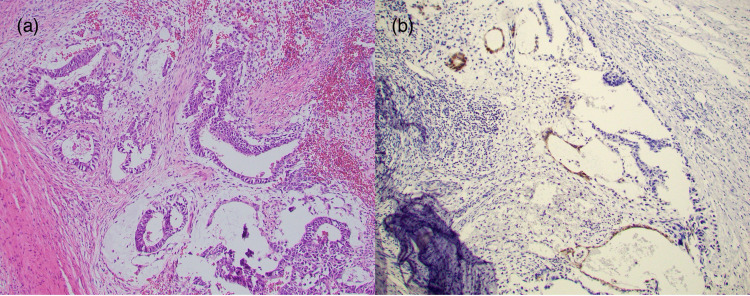
Yolk Sac Component in Left Testicular Tumor (a) H&E stain of the yolk sac tumor component in the patient’s mixed testicular germ cell tumor showing macrocystic pattern of columnar cells. (b) Immunohistochemical stain showing glypican-3 expression on a section of the yolk sac tumor component.

The patient declined sperm banking before surgery. He underwent left radical orchiectomy in April 2020. Testicular pathology (Figure 3) showed a 2.2 cm mixed germ cell tumor composed of post-pubertal type teratoma (85%), embryonal carcinoma (10%) (Figure 4), and yolk sac tumor (5%) (Figure 5) in addition to a separate tumor focus of classic seminoma, sized 0.8 cm, and germ cell neoplasia in situ. Computerized tomography (CT) scan of the chest/abdomen/pelvis with contrast showed no evidence of metastasis. Tumor markers after left orchiectomy were: AFP 3.4 ng/mL, HCG <1 mIU/mL, LDH 179 IU/L. Final staging was pT1N0M0.

**Figure 6:**
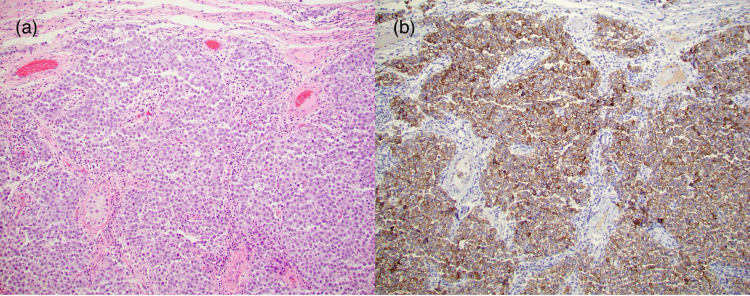
Right Testicular Tumor Histology (a) H&E stain of the patient’s right testicular tumor shows classic seminoma with diffuse sheets of tumor cells interrupted by fibrous septa containing lymphocytes. (b) Immunohistochemical stain showing CD117 expression on a section of the seminomatous tumor.

In May 2020, the patient underwent repeat scrotal ultrasound of his right testis, which showed an increase in size of both testicular masses. The size of the mass in the mid inferior portion of the right testis was 3.5 cm X 2.9 cm X 2.2 cm. The second mass was 1.9 cm X 1.8 cm x 1.3 cm on the lateral portion of the right testis. Both the masses were hypoechoic and showed peripheral and internal vascularity. The patient underwent right radical orchiectomy in June 2020. Final pathology (Figure 6) showed two foci of classic seminoma (3 cm, 2.1 cm) germ cell neoplasia in situ. CT abdomen and pelvis with contrast and chest X-ray were unremarkable. Tumor markers after right orchiectomy were AFP 3 ng/mL, HCG <1 mIU/mL, and LDH 188 IU/mL. Final staging was pT1bN0M0. At present, he uses testosterone gel. He is on active surveillance per guidelines and monitoring of testosterone levels. At this time, the patient remains in remission at 18 months per imaging and tumor markers.

## Discussion

The 2016 WHO classification scheme defines the two major entities of testicular germ cell tumors (TGCTs) as non-germ cell neoplasia in situ related (non-GCNIS) and germ cell neoplasia in situ related (GCNIS) tumors. The non-GCNIS related tumors can be subdivided into types I and III, while the GCNIS-related tumors are type II TGCTs [9]. Type I TGCTs are prepubertal teratomas and yolk sac tumors, originating from pre-erased or partially erased embryonic germ cells or embryonic stem cells. Type II TGCTs, which occur most frequently, are malignant and include seminomas and non-seminomas. Type III TGCTs include spermatocyte tumors arising from fully erased and paternally imprinted germ cells. Age of clinical presentation plays a major role in identifying TGCT subtype, with type I manifesting prior to puberty and types II and III manifesting after puberty [9]. Our case primarily involves type II TGCT, namely classic seminoma in the right testis and mixed germ cell tumors in the left testis - teratoma, seminoma, and germ cell neoplasia in situ.

Bilateral testicular tumors are extremely rare, comprising roughly 1-3% of all TGCTs [3]. The majority of these bilateral tumors are metachronous. Bilateral synchronous testicular tumors are estimated to account for 10% of the bilateral tumors, and are predominantly of identical histology, typically with bilateral seminoma [2]. Fewer than 100 cases of discordant histological presentations of bilateral testicular tumors have been described in the literature (Table 1). The first case of synchronous bilateral TGCT with different histology was discovered in 1954 [4]. The patient was found to have seminoma in his right testis and teratoma in his left testis and underwent bilateral orchiectomy. A review of the literature by Coli et al. [5] in 2003 documented 42 cases of bilateral synchronous TGCTs of different histopathology, in addition to their own case. In 2017, Campobasso et al. [6] compiled another review of literature identifying 73 cases of synchronous bilateral testicular cancer, five of which were previously included by Coli et al. Excluding those and two cases of bilateral Leydig cell tumor, 62.1% of these patients had concordant histology, the majority of which were bilateral seminoma. The remaining 37.9% presented with discordant histologies of varying stages - 54.5% (12/22) stage I, 13.6% (3/22) stage II, and 31.8% (7/22) stage III. Of the discordant cases, three had specified histologies - one non-seminoma/epidermoid cyst and two seminoma/embryonal carcinoma. The rest were not specified. More recently in 2019, Lu et al. [7] identified 11 patients out of 118 TGCT cases who presented with bilateral tumors of discordant histology. Since then, a handful of individual cases have been reported. In one case, a 20-year-old man presented with mixed germ-cell tumor with seminoma and embryonal carcinoma in his right testis and embryonal carcinoma and intratubular germ-cell neoplasia unclassified infiltrating the surgical margins in his left testis [2]. The patient underwent a right radical orchiectomy and left testis-sparing surgery with concomitant onco-testicular sperm extraction initially, before eventually receiving a left orchiectomy with adjuvant chemotherapy consisting of bleomycin, etoposide, and cisplatin (BEP). The patient remained cancer free at 18-month follow-up. Another report describes the case of a young otherwise healthy man who presented with bilateral discordant histology, specifically a right testicular seminoma and left testicular mixed germ cell tumor composed of seminoma, yolk sac tumor and embryonal carcinoma [3]. This patient remained cancer-free six months following treatment with one round of BEP.

**Table 1:** 91 Cases of Bilateral Synchronous Testicular Germ Cell Tumors with Discordant Histology BO: bilateral orchiectomy; UO: unilateral orchiectomy; RPLND: retroperitoneal lymph node dissection

	Coleman PN, McKeown KC (1954) [4]	Coli A et al. (2003) [5]	Campobasso D et al. (2017) [6]	Lu N et al. (2019) [7]	Symeonidis EN et al. (2021) [2]	Rostami G et al. (2020) [3]
No. Cases of Bilateral Synchronous TGCT with discordant histology (excluding repeats)	1	42	20	11	16	1
Treatment:						
BO	1/1 (100%)	23/42 (54.8%)	16/20 (80%)	0/11 (0%)	15/16 (93.8%)	1/1 (100%)
UO	0/1 (0%)	2/42 (4.8%)	4/20 (20%)	0/11 (0%)	0/16 (0%)	0/1 (0%)
RPLND	0/1 (0%)	12/42 (28.6%)	5/20 (25%)	0/11 (0%)	1/16 (6.25%)	0/1 (0%)
Radiation Therapy	0/1 (0%)	6/42 (14.3%)	0/20 (0%)	0/11 (0%)	1/16 (6.25%)	0/1 (0%)
Chemotherapy	0/1 (0%)	19/42 (45.2%)	13/20 (65%)	0/11 (0%)	11/16 (68.8%)	1/1 (100%)
Unknown/Not Specified	0/1 (0%)	14/42 (33.3%)	0/20 (0%)	11/11 (100%)	5/16 (3.13%)	0/1 (0%)
Outcome:						
No Evidence of Disease	0/1 (0%)	21/42 (50%)	19/20 (95%)	11/11 (100%)	11/16 (68.8%)	1/1 (100%)
Evidence of Relapse	0/1 (0%)	3/42 (7.1%)	1/20 (5%)	0/11 (0%)	3/16 (18.8%)	0/1 (0%)
Died of Disease	0/1 (0%)	3/42 (7.1%)	1/20 (5%)	0/11 (0%)	0/16 (0%)	0/1 (0%)
Unknown/Not Specified	1/1 (100%)	16/42 (38.1%)	0/20 (0%)	0/11 (0%)	2/16 (12.5%)	0/1 (0%)

The exact pathogenesis behind the development of synchronous bilateral TGCT remains to be elucidated. Previously there was disagreement whether separate oncogenic mechanisms occur in both testes simultaneously or contralateral metastasis occurs. It was first proposed by Matsushima et al. [10] in 1987 that each lesion of bilateral TGCT of identical tissue type is developed independently as a primary tumor given the absence of lymphatic and vascular connections between the two testes. Researchers have suggested the presence of variable activation, de-repression, and repression of differentiation genes to play a role in the development of these tumors with different histological subtypes [11]. It has since been largely accepted that bilateral synchronous tumors of different histology follow a similar mechanism and occur as the development of two independent primary tumors rather than as a metastasis from one testis to the other. Accordingly, both tumors in our patient’s case were independently staged.

Bilateral synchronous testicular tumors are typically caught at an early stage and thus carry a good prognosis. In 2005, Hentrich et al. [8] reported nine out of 14 patients with synchronous TGCT to have no evidence of disease at a median follow-up of 37 months. Based on a systematic review of bilateral TGCT cases between 1991 and 2011, the five-year survival rate of synchronous TGCT was estimated to be 88% [12]. However, it should be noted that synchronous TGCTs in the review were found to be correlated with more advanced disease and less favorable survival rates than their metachronous counterparts. Survival analysis of the synchronous tumor cases further revealed that discordant histology negatively impacted overall survival and disease-specific survival rates. A likely explanation consistent with the findings of previously reported studies may be that patients with bilateral seminomas generally present at a lower stage at diagnosis while patients with bilateral non-seminomatous TGCTs present at higher stages at diagnosis and thus have poorer prognoses.

Treatment for patients with synchronous bilateral TGCT of different histology should reflect standard management of unilateral testicular carcinoma. Bilateral orchiectomy is considered the definitive procedure for pathological diagnosis and local control. Following bilateral orchiectomy, several management options, including radiotherapy, chemotherapy, and active surveillance, can be utilized depending on the stage and histology of the tumors. Specifically, the therapeutic approach is determined by the most malignant component between the bilateral tumors [13]. In our case, our patient was determined to have stage 1 pure seminoma tumors in the right testis and a stage 1 mixed germ cell tumor in the left testis. As such, our regimen selection was tailored to the more malignant left-sided tumor consisting of a teratomatous component. Given this left-sided tumor was stage 1 of low risk, we opted for active surveillance following bilateral orchiectomy. The patient remains in remission at 18-month follow-up.

## Conclusions

Bilateral primary synchronous TGCTs typically present with identical histology in each tumor. It is very uncommon for bilateral testicular tumors to present with different histopathology. Our patient presented with a case of primary synchronous bilateral testicular cancer with discordant histology in each testis. Despite the rarity of this condition, its treatment mirrors standard management of unilateral testicular carcinoma, with the added element of prioritization of one tumor over the other. It is important for clinicians to treat bilateral TGCT based upon the most malignant component.
